# The association between periodontal disease and metabolic syndrome among outpatients with diabetes in Jordan

**DOI:** 10.1186/s40200-015-0192-8

**Published:** 2015-08-16

**Authors:** Rola Alhabashneh, Yousef Khader, Zaid herra, Farah Assad

**Affiliations:** Preventive Department-Periodontics, College of Dentistry, Jordan University of Science and Technology, PO Box: 3030, Irbid, 22110 Jordan; Department of Community Medicine, Jordan University of Science and Technology, PO Box: 3030, Irbid, 22110 Jordan

**Keywords:** Periodontitis, Diabetes, Metabolic syndrome

## Abstract

**Background:**

To date, conflicting results have been reported about the association between metabolic syndrome (MetS) and periodonttitis.

**Methods:**

Two hundred and eighty patients with type 2 diabetes were recruited from outpatients visiting diabetes clinics in Islamic Hospital, Amman-Jordan. The oral hygiene and the periodontal status of all teeth, excluding third molars, were assessed using the plaque index of Silness and Löe, the gingival index of Löe and Silness, probing pocket depth (PPD), and clinical attachment level (CAL). Data were analyzed using the general linear model multivariate procedure with average PPD, average CAL, percent of teeth with CAL ≥3 mm, and percent of teeth with PPD ≥3 mm as outcome variables and diabetes, MetS and its individual components as predictors.

**Results:**

Overall, 83.2 % of patients with diabetes had MetS. In the multivariate analysis, patients with MetS had a significantly more severe periodontitis, as measured by average PPD and average CAL (*P* < 0.005). The extent of periodontitis, as measured by the percent of teeth with CAL ≥3 mm and the percent of teeth with PPD ≥3 mm, was also significantly greater among patients with MetS (*P* < 0.005). As the number of metabolic components additional to diabetes increased, the odds of having periodontitis increased, and the odds were greatest when all the components additional to diabetes were present (OR = 10.77, 95 % CI: 2.23 -51.95).

**Conclusion:**

Patients with MetS displayed more severe and extensive periodontitis. Having other MetS components additional to diabetes increased the odds of having periodontitis.

## Introduction

Metabolic syndrome (MetS) is a complex collection of components that are thought to arise from a visceral fat-type obesity involving hypertension and abnormal glucose and lipid metabolism [1]. The prevalence of MetS increases with age and varies with ethnicity and race [2]. The prevalence of MetS among adults in USA is 22.9 % [3], which is comparable to its prevalence among Canadian adult population (19.1 %) [4]. Higher MetS prevalence rates have been observed in the Eastern Mediterranean countries and Arab populations [5–9]. In Jordan, the prevalence of MetS among adults was alarmingly high (36.6 %) [10]. Lower prevalence of MetS has been found among European adults [11].

Periodontal disease is a group of infectious diseases triggered by periodontopathogens [12]. It is considered the most common chronic infection worldwide and in the US [13, 14]. Periodontitis and MetS are multi-factorial diseases sharing a common inflammatory pathway. Many people with MetS have a low grade systemic inflammation which is reported by elevated levels of inflammatory mediators such as C-reactive protein (CRP), interleukin-6 (IL-6), and tumor necrotic factor- α (TNF-α). Moreover, people who have periodontitis, which is a chronic inflammation, also have elevated levels of inflammatory markers (CRP, IL-6 and TNF-α) [15, 16]. There is growing evidence suggesting an association between periodontal disease and MetS components since positive correlation between obesity, hypertension, hyperglycemia and hyperlipidemia with periodontal disease has been reported [17, 18]. MetS has been shown to be associated with the prevalence, extent and severity of periodontal disease [15, 19–21] and a recent systematic review concluded that subjects affected by MetS are nearly twice more likely to have periodontitis than the rest of the population [22]. Furthermore, periodontal disease has effects on the glycemic control [23] and lipid metabolism [24].

This study is built on the concept that the three diseases (MetS, periodontal disease and diabetes mellitus) share common pathogenesis. The aim of this study was to assess the association between MetS and periodontal disease among patients with type 2 diabetes in Jordan.

## Methods

### Study population and sampling

The study was approved by the University Ethical Committee at Jordan University of Science and Technology. The participants were recruited from outpatients visiting the diabetes clinics in Islamic Hospital Amman-Jordan, over a period of six months between June-November 2011. All participants were interviewed and examined after they signed a written consent form. Patients were selected using systematic random sampling technique by choosing every third patient (i.e. patients registered with numbers such as 3, 6, 9, 12…) attending the clinics. About half of the interviewed patients met the inclusion criteria. Patients with a history of a systemic condition or medication use that might influence the severity of periodontitis were excluded (i.e. patients with a history of thyroid diseases, chronic renal problems and connective tissue diseases). Pregnant women and patients who were edentulous and those who had undergone periodontal treatment within the preceding 6 months were also excluded.

The power of this study to detect the association between MetS and periodontal disease given our sample size was calculated. To detect a minimal odds ratio of 3 at alpha level of 5 % in a sample that included 233 patients with MetS and assuming that 14 % of patients without MetS had periodontal disease, the power was calculated as 99 %. The power calculations were performed using Sample size online calculator (http://sampsize.sourceforge.net/iface/s3.html).

### Data collection

Data were collected by a questionnaire answered by patients through personal interview and clinical examination. The questionnaire included questions about socio-demographic variables, medical history, smoking history, previous dental history, oral hygiene practice and clinical findings associated with periodontal examination. Participants were assured of the confidentiality of all obtained information and that collected data will only be used for scientific purposes.

### Diabetes mellitus

Only patients with type 2 diabetes were included in this study. Patients were diagnosed with diabetes mellitus if they had fasting plasma glucose (FPG) ≥ 100 mg/dl or if they were taking medications for type 2 diabetes mellitus. Control of diabetes mellitus was investigated through glycated hemoglobin test (HbA1c). The FPG and HbA1c values were taken from recent tests included in patients’ files.

### Hypertension

Hypertension was defined as systolic blood pressure ≥ 130 mmHg or diastolic blood pressure ≥ 85 mmHg, or under treatment of previously diagnosed hypertension as reported in patients’ medical records. Blood pressure was measured by a qualified nurse using a mercury sphygmomanometer on the same day of periodontal examination.

### Hyperlipidemia

The diagnosis of hyperlipidemia was made by an endocrinologist based on the measurements of triglycerides (TG) and high density lipoproteins (HDL): TG ≥ 150 mg/dl and/or (HDL cholesterol level < 40 mg/dl in males and < 50 mg/dl in females), or if the patient was currently on specific treatment for lipid abnormalities.

### Obesity

The participants were diagnosed as obese by measuring the waist circumference (WC), using a circumference measuring tape. The readings were rounded to the nearest centimeter. The diagnosis was made if the waist circumference was ≥ 94 cm for men and ≥ 80 cm for women. The weight was measured using a mechanical flat scale to the nearest kilogram, and height was measured to the nearest centimeter using a measuring rod. All these measurement were taken at the same day of examination, with the participants wearing light clothing and no shoes.

#### MetS definition

MetS was defined according to the International Diabetes Federation (IDF) definition [25]. According to the IDF definition, a person was defined as having MeS if he or she had central obesity (defined as waist circumference ≥94 cm for men and ≥80 cm for women, with ethnicity specific values for other groups) plus any two of the following four factors: 1) Raised TG level: ≥150 mg/dL (1.7 mmol/L), or specific treatment for this lipid abnormality, 2) Reduced HDL cholesterol: <40 mg/dL (1.03 mmol/L) in males and <50 mg/dL (1.29 mmol/L) in females, or specific treatment for this lipid abnormality, 3) Raised blood pressure: systolic BP ≥130 or diastolic BP ≥85 mm Hg, or treatment of previously diagnosed hypertension, 4) Raised fasting plasma glucose (FPG) ≥100 mg/dL (5.6 mmol/L), or previously diagnosed type 2 diabetes.

### Periodontal clinical examination

The oral hygiene and the periodontal status of all teeth, excluding third molars, were assessed using, plaque index (PI) of Silness & Löe (1964) [26], probing pocket depth (PPD), and clinical attachment loss (CAL). Sterile dental mirrors and explorers were used to assess plaque accumulation and gingival status. Hu-Friedy periodontal probes with Williams’s markings (Diatech, Switzerland) were used to measure PPD and CAL. All teeth (excluding third molars), and six surfaces of each studied tooth (mesio-facial, mid-facial, disto-facial, mesio-lingual, mid-lingual, and disto-lingual) were assessed and scored for PI, GI, PPD and CAL. Plaque index was scored as follows: 0 = no plaque/debris 1 = A film of plaque adhering to the free gingival margin and adjacent area of the tooth. 2 = Moderate accumulation of soft deposit s within the gingival pocket, or the tooth and gingival margin which can be seen with the naked eye. 3 = Abundance of soft matter within the gingival pocket and/or on the tooth and gingival margin. The highest score of each reading was taken into consideration. Data were recorded on periodontal examination form.

Disease extent was defined by the percentage of deep pockets. The mean of the deepest reading for PPD and CAL was calculated within each mouth by dividing the value of deepest pockets on the total number of deep pockets. Periodontitis was defined as presence of four or more teeth with highest reading of PPD ≥ 3 mm and CAL ≥ 3 mm [27].

### Reliability of questionnaire and periodontal examination

Clinical examinations were performed by one skilled examiner (RA). Before the beginning of the study, the measurement reliability was determined on the basis of examinations performed on 10 patients with severe periodontitis. Of the replications, 98 % were within 1 mm for PDs, and 97 % were within 1 mm for CALs.

### Data management and analysis

The Statistical Package for Social Sciences software (SPSS Inc., version 15, Chicago, IL, USA) was used for data processing and data analysis. Characteristics of subjects’ variables were described using frequency distribution for categorical variables and mean and standard deviation for continuous variables. The Chi^2^ test was used to assess the association between categorical variables.

The multivariate analysis of the association between different disease status and periodontal parameters (average PPD, average CAL) and percentage of teeth with (PPD ≥3 mm, PPD ≥4 mm, PPD ≥5 mm, PPD ≥6 mm, CAL ≥3 mm, CAL ≥4 mm, CAL ≥5 mm and CAL ≥6 mm) was conducted using the General Linear Model procedure. Multivariate binary logistic regression was conducted to determine the association between each disease group (i.e. group of patients with MetS and group of patients with diabetes) and the prevalence of periodontal disease after adjusting for important variables. All variables that were significantly associated with periodontal disease constituted the best regression model. The association between obesity, hypertension, and hyperlipidemia, with diabetes and periodontal disease was tested, in separate models, for each disease group after adding that indicator to the best model. The association between diabetes, the increasing number of MetS components and periodontal disease was tested too. The statistical significance of the two-way interactions between independent variables was assessed with the use of forward stepwise regression.

The two-way interaction terms, one at a time, were added in the model containing all the main effects and were assessed for their significance using the likelihood ratio test. Crude odds ratios (ORs) and their 95 % confidence interval (CI) were calculated. A p-value of <0.05 was considered statistically significant.

## Results

This study included a total of 280 patients with type 2 diabetes mellitus. Overall, 83.2 % of patients with diabetes had MetS. The patients aged between 21 and 80 years with a mean (±SD) age of 53.8 (±9.6) years. Socio-demographic, dental, anthropometric, clinical, and relevant characteristics of the participants are shown in Tables 1 and 2. About half (50.7 %) of patients were males and 49.3 % were females. About one third of patients (32 %) had university level education. Almost 30 % of patients reported brushing their teeth twice daily. Nearly half (51.8 %) of patients had duration of diabetes of ≤ 5 years, 28 % were taking insulin therapy, and 93.2 % were on oral hypoglycemic medications. Only 21.8 % of patients were well controlled (HbA1c ≤6.5 %). The majority (87.5 %) of the participants were obese, 87.1 % had elevated blood pressure, 65 % had increased triglycerides level (≥150 mg/dl), and 46.1 % had low HDL level.

**Table 1:** Socio-demographic, dental and relevant characteristics of 280 patients with type 2 diabetes

Variable	n (%)
Gender:	
Male	142 (50.7)
Female	138 (49.3)
Age (years):	
≤45	56 (20.0)
46–55	107 (38.2)
>55	117 (41.8)
Family income (JD):	
≤500 JD	168 (60)
>500	112 (40)
Education:	
Elementary	107 (38.2)
Secondary	86 (30.7)
University	87 (31.1)
Residency area:	
Urban	271 (96.8)
Rural	9 (3.2)
Smoking:	
Non-smoker	185 (66.1)
X-smoker	34 (12.1)
Smoker	61 (21.8)
Teeth brushing:	
Infrequent	87 (31.1)
Once/day	109 (38.9)
Twice/day	84 (30.0)
Having fixed prosthesis	186 (66.4)
Number of missing teeth:	
0	53 (18.7)
1–7	120 (43.0)
8–18	107 (40.3)
Periodontitis	111 (39.6)

**Table 2:** Anthropometric, clinical and relevant characteristics of 280 patients with type 2 diabetes

Variables	n (%)
Diabetes duration (years):	
≤5	145 (51.8)
>5	135 (48.2)
HbA1c (%):	
≤6.5	61 (21.8)
>6.5	219 (78.2)
Diabetes treatment:	
On insulin	78 (27.9)
On oral hypoglycemic drugs	261 (93.2)
Increased Waist circumference	245 (87.5)
Elevated blood pressure	244 (87.1)
Increased triglycerides level	182 (65)
Low HDL	129 (46.1)
Metabolic syndrome (MetS)	233 (83.2)

Table 3 illustrates the extent and severity of periodontal disease according to MetS after adjusting for average PI, age, family income, residency area, smoking, teeth brushing and number of missing teeth. Patients with MetS had a significantly higher mean PPD (*P* < 0.005) and CAL (*P* = 0.001). The extent of periodontal disease was also significantly higher in patients with MetS as measured by average percent of teeth with the deepest CAL ≥3 mm (*P* < 0.005), CAL ≥4 mm (*P* < 0.005), and CAL ≥5 mm (*P* < 0.005).

**Table 3:** Extent and severity of periodontal disease according to metabolic syndrome (MetS) among 280 patients with type 2 diabetes

Variable	Metabolic syndrome		*P*-value*
	No (n = 47)	Yes (n = 233)	
	Mean (SD)	Mean (SD)	
Average Probing Pocket Depth (PPD)	1.29 (0.32)	1.77 (0.42)	<0.005
Average Clinical Attachment Level (CAL)	1.52 (1.34)	2.86 (1.59)	0.001
Average percent of teeth with deepest:			
PPD ≥ 3	1.00 (2.83)	11.08 (21.89)	0.038
PPD ≥ 4	0.33 (1.36)	7.03 (20.77)	0.159
PPD ≥ 5	0.0 (0.0)	5.24 (20.43)	0.297
PPD ≥ 6	0.0 (0.0)	4.40 (20.30)	0.392
Average percent of teeth with deepest:			
CAL ≥ 3	13.25 (23.24)	54.54 (28.13)	<0.005
CAL ≥ 4	10.44 (17.79)	40.13 (30.05)	<0.005
CAL ≥ 5	3.02 (6.93)	24.03 (24.75)	<0.005
CAL ≥ 6	1.89 (5.09)	14.99 (22.76)	0.160

*Adjusted for average PI, age, family income, residency area, smoking, teeth brushing, and number of missing teeth

Table 4 demonstrates the extent and severity of periodontal disease according to the number of metabolic abnormalities in the multivariate analysis. As the number of metabolic abnormalities in addition to diabetes increased; the severity of periodontal disease, as measured by average PPD and average CAL, significantly increased. The increasing number of components of MetS was significantly associated with higher extent of periodontal disease as measured by the average percent of teeth with CAL ≥3 mm, ≥4 mm, ≥5 mm, and ≥6 mm.

**Table 4:** Extent and severity of periodontal disease according to the number of metabolic syndrome (MetS) components among 280 patients with type 2 diabetes

	The number of MetS components additional to diabetes mellitus				
	One component *n* = 31 Mean (SD)	Two components *n* = 52 Mean (SD)	Three components *n* = 115 Mean (SD)	Four components *n* = 82 Mean (SD)	*P*-value*
Average Pocket Probing Depth (PPD)	1.25 (0.33)	1.71 (0.33)	1.7 (0.43)	1.85 (0.46)	<0.005
Average Clinical Attachment Level (CAL)	1.11 (1.22)	2.33 (1.3)	2.85 (1.6)	3.1 (1.65)	<0.005
Average percent of teeth with deepest:					
PPD ≥ 3	0.91 (2.61)	6.58 (10.67)	10.40 (23.01)	12.95 (23.64)	0.459
PPD ≥ 4	0.38 (1.53)	1.82 (5.17)	7.21 (22.43)	8.76 (22.36)	0.345
PPD ≥ 5	0.0 (0.0)	0.74 (18.77)	6.03 (22.31)	5.98 (21.77)	0.437
PPD ≥ 6	0.0 (0.0)	0.07 (0.5)	5.32 (22.32)	5.00 (21.66)	0.433
Average percent of teeth with deepest:					
CAL ≥ 3	9.82 (15.73)	39.54 (28.22)	52.94 (29.46)	59.5 (28.45)	<0.005
CAL ≥ 4	6.95 (13.07)	27.27 (27.04)	39.01 (28.72)	45.34 (32.2)	<0.005
CAL ≥ 5	2.77 (7.54)	16.32 (20.83)	21.66 (24.11)	28.22 (26.23)	0.005
CAL ≥ 6	1.04 (3.34)	6.99 (9.93)	13.08 (20.61)	20.48 (27.99)	0.004

*Adjusted for average PI, age, family income, residency area, smoking, teeth brushing, and number of missing teeth

The prevalence of periodontal disease according to MetS and its components is shown in Table 5. The prevalence of periodontal disease was higher among those who had MetS compared to those who did not have MetS (44.6 % vs. 14.9 %). The prevalence of periodontal disease in patients with diabetes was significantly increased as the number of metabolic components increased (*P* < 0.005). Diabetes mellitus was associated with significantly higher prevalence of periodontal disease when accompanied with increased waist circumference (*P* = 0.011), increased triglycerides (*P* = 0.005), and decreased HDL (*P* = 0.001), but not when accompanied with hypertension (*P* = 0.119).

**Table 5:** Prevalence of periodontal disease among 280 patients with diabetes mellitus according to metabolic syndrome (MetS) and its components

	Periodontitis		*p*-value
	Non (%)	Yesn (%)	
Metabolic syndrome (MetS)			<0.005
No	40 (85.1)	7 (14.9)	
Yes	129 (55.4)	104 (44.6)	
Number of metabolic components additional to diabetes mellitus			
1 component	29 (93.5)	2 (6.5)	<0.005
2 components	32 (61.5)	20 (38.5)	<0.005
3 components	72 (62.6)	43 (37.4)	<0.005
4 components	36 (43.9)	46 (56.1)	<0.005
Waist circumference			0.011
Normal	28 (80)	7 (20)	
Increased	141 (57.6)	104 (42.4)	
Blood pressure			0.119
Normal	26 (72.2)	10 (27.8)	
Elevated blood pressure	143 (58.6)	101 (41.4)	
Triglycerides			0.005
Normal	70 (71.4)	28 (28.6)	
Increased	99 (54.4)	83 (45.6)	
HDL			0.001
Normal	105 (69.5)	46 (30.5)	
Low	64 (49.6)	65 (50.4)	

Model I in Table 6 shows that patients with MetS were almost 3 times (OR = 3.28, 95 % CI: 1.3-8.3) more likely to have periodontal disease compared to patients without MetS. In model II, patients with diabetes were about 6 times (OR = 5.73, 95 % CI: 1.15-28.59) more likely to have periodontal disease if they had two more components of MetS compared to those who had one additional component only. Patients who had all the other components of metabolic syndrome had much higher odds of having periodontal disease (OR = 10.77, 95 % CI: 2.23-51.95) compared to those who had one additional component only. In model III, of the individual components of MetS, only HDL level was significantly associated with increased odds of having periodontal disease (OR = 1.99, 95 % CI: 1.15-3.46).

**Table 6:** Multivariate analysis of association between metabolic syndrome (MetS) and its components with periodontal disease in separate models among 280 patients with diabetes mellitus

	Odds ratio (95 % confidence interval) of having periodontitis	*p*-value*
Model I		
Metabolic syndrome (MetS)		
No	1	
Yes	3.28 (1.30, 8.30)	0.012
Model II: Number of MetS additional to diabetes		
1 component	1	
2 components	5.73 (1.15, 28.59)	0.03
3 components	4.82 (1.03, 22.65)	0.046
4 components	10.77 (2.23, 51.95)	0.003
Model III: Individual components of MetS		
Waist circumference		
Normal	1	
Increased	1.74 (0.63, 4.79)	0.286
Blood pressure		
Normal	1	
Elevated	1.12 (0.43, 2.90)	0.812
Triglycerides		
Normal	1	
Increased	1.41 (0.77, 2.60)	0.268
HDL		
Normal	1	
Low	1.99 (1.15, 3.46)	0.004

*In the three models, the associations between periodontal disease and metabolic syndrome, individual components, and their combinations were adjusted for average PI, age, family income, residency area, smoking, teeth brushing, and number of missing teeth

## Discussion

This study showed a positive association between periodontitis and MetS. Periodontitis was more common and severe in diabetic patients with MetS in Jordan compared to patients with no MetS. As the number of metabolic components additional to diabetes increased the severity and extent of periodontal disease increased.

Our study is the first in the Middle East that explores the association between MetS and periodontal disease among patients with diabetes. Other studies compared periodontal disease between patients with MetS and patients without MetS only without investigating the effect of individual components of MetS.

The findings of this study are consistent with the study conducted by Khader et al. (2008) [19] in Jordan and are in support of other studies‘ findings [20, 21]. Shimazaki et al. (2007) [28] reported that MetS is associated with increased risk of periodontal disease among Japanese women. On the other hand, Morita et al. (2010) [16] concluded that presence of periodontal pockets was associated with a positive conversion of MetS components, suggesting that preventing periodontal disease may prevent MetS. These studies may propose a two way relationship. However, longitudinal and interventional studies should be performed to investigate such a relationship.

In this study when each component additional to diabetes was analyzed separately, increased waist circumference (WC) and low HDL level were associated with significantly higher extent and severity of periodontal disease. Increased triglycerides (TG) level was associated with significant increase in the extent but not severity of periodontal disease, while hypertension was not significantly associated with either extent or severity of periodontal disease. This was comparable with the results reported by Shimazaki et al. (2007) [28], as increased WC, low HDL and fasting blood sugar had significant relationships with periodontal disease.

In contrast, Kushiyama et al. (2009) [20] reported that of the five MetS components, only high blood pressure and low HDL were each significantly associated with deep probing depths.

When the extent and severity of periodontal disease were analyzed according to the number of MetS components in multivariate analysis, the number of components additional to diabetes increased was associated with increased extent and severity of periodontal disease. This finding was consistent with results of other studies [15, 19, 20, 28]. It is important to mention that these studies were designed to compare periodontal disease between patients with MetS and patients without MetS, but not in patients who had diabetes and MetS. In this study, the effect of diabetes on periodontal disease was the principal factor to which the effect of other components was added, while the other studies did not take into consideration the well established relationship between diabetes and periodontal disease.

In our study, obesity was significantly associated with higher extent and severity of periodontal disease. This finding was consistent with the findings of another study in Jordan by Khader et al. (2009) [29]. Low HDL level was strongly associated with increased extent and severity of periodontal disease, while elevated TG levels were associated with significant increase in average CAL (severity) and percent of teeth with deepest CAL ≥ 3, ≥ 4 mm (extent). These findings are in agreement with the study conducted by Fentoğlu et al. (2009) [30]. However, Saxlin et al. (2008) [31] found no association between level of serum lipids and periodontal disease in normal-weight subjects, while obese subjects with high TG levels, and/or low HDL levels could be at high risk of periodontal disease.

Diabetic patients who had MetS had significantly higher prevalence of periodontal disease than those without MetS. This finding confirmed the results of Li et al. (2009) [15] and Kushiyama et al. (2009) [20]. In this study the prevalence of periodontitis in patients with diabetes was significantly increased as the number of MetS components increased. This was similar to other studies; Kushyiama et al. (2009) [20] concluded that subjects with three, four or five components of MetS exhibited a significantly higher prevalence of periodontal disease and Li et al. (2009) [15] found that prevalence of periodontal disease was less in subjects bearing fewer than two of metabolic components. Also, Hasegawa et al. (2004) [32] observed that Japanese urban women who had three or more components had a higher prevalence of periodontal disease than those who had less than two components.

In this study only low HDL level was significantly associated with increased odds of having periodontal disease in the multivariate analysis. Alike, Shimazaki et al. (2007) [28] reported that of the five components of MetS, low HDL level had the highest odds ratio and was statistically significant for a greater PPD and CAL. Similarly, Nibali et al. (2007) [33] reported that periodontitis exhibited a significant association with low HDL, high LDL, and elevated fasting blood sugar.

In conclusion, patients with MetS displayed more severe and extensive periodontitis. Having other MetS components additional to diabetes increased the odds of having periodontitis. Given the study design we used, our findings reflect the cumulative and additive effects of metabolic abnormalities on the risk of periodontal disease. Longitudinal and interventional studies are needed to confirm the study findings.
