# Erratum to: The association between periodontal disease and metabolic syndrome among outpatients with diabetes in Jordan

**DOI:** 10.1186/s40200-015-0207-5

**Published:** 2015-11-13

**Authors:** Rola Alhabashneh, Yousef Khader, Zaid Herra, Farah Asa’ad

**Affiliations:** Preventive Department-Periodontics, College of Dentistry, Jordan University of Science and Technology, Irbid, 22110 Jordan; Department of Community Medicine, Jordan University of Science and Technology, Irbid, 22110 Jordan

The original version of this article [1] unfortunately contained a mistake. The spelling of Farah Asa’ad’s name was incorrect. The original article has also been updated to reflect this change.
